# Leadership styles and personal values of professors at a school of nursing

**DOI:** 10.1590/0034-7167-2023-0333

**Published:** 2024-08-26

**Authors:** Chennyfer Dobbins Abi Rached, Bianca Batista de Siqueira, Bruna Franco Massa, Caroline da Silva Fonseca Paulo, Sofia de Souza Cruz

**Affiliations:** I Universidade de São Paulo. São Paulo, São Paulo, Brazil

**Keywords:** Leadership, Faculty, Faculty, Nursing, Education, Education, Nursing, Liderazgo, Docentes, Docentes de Enfermería, Educación, Educación en Enfermería

## Abstract

**Objectives:**

to identify professors’ leadership styles and personal values.

**Methods:**

a quantitative, descriptive-exploratory study. Population was made up of professors in doctoral category 1 at a public university in the state of São Paulo. Data collection took place from June to August 2021. Sociodemographic characterization was extracted, and the Leadership Team Values Assessment was applied. Data were analyzed using measures of central tendency.

**Results:**

population included 13 professors. The level that represents authentic leadership was the most prevalent. The commitment, positive attitude and trust values stood out. The level portrayed by visionary leadership was the least identified.

**Conclusions:**

professors’ personal values provide a theoretical basis for guiding and analyzing professors’ leadership styles. Leadership in the educational context must be recognized and studied to promote a more comprehensive and effective approach to developing and improving educational leaders.

## RESUMEN

**Objetivos:**

identificar los estilos de liderazgo de profesores universitarios y sus valores personales.

**Métodos:**

estudio cuantitativo, descriptivo-exploratorio. Población compuesta por profesores universitarios de la categoría 1 de doctorado de una universidad pública del estado de São Paulo. La recopilación de datos se realizó de junio a agosto de 2021. Se extrajo la caracterización sociodemográfica y se aplicó el instrumento *Leadership Team Values Assessment*. Los datos se analizaron utilizando medidas de tendencia central.

**Resultados:**

la población estuvo integrada por 13 docentes. El nivel que representa un liderazgo auténtico fue el más prevalente. Se destacaron los valores compromiso, actitud positiva y confianza. El nivel retratado por el liderazgo visionario fue el menos identificado.

**Conclusiones:**

los valores personales de los docentes proporcionan una base teórica para orientar y analizar los estilos de liderazgo docente. El liderazgo en el contexto educativo debe ser reconocido y estudiado para promover un enfoque más integral y eficaz para desarrollar y mejorar a los líderes educativos.

## INTRODUCTION

Academic literature has a broad theoretical framework regarding the study of human values. This interest is due to the conception, reiterated by several authors, that values are predictive of human behavior, whether in the personal, collective, family, or professional spheres. Therefore, it is possible to explore various individual, collective and social aspects based on the understanding of human values^(1)^.

Academic studies related to values were not limited to clarifying the term, but proposed ways of assessing values, since the analysis of a leader’s personal values says a lot about their motivations and leadership style. Some validated scales for assessing valuesstand out, such as the Portrait Questionnaire Value (PQV), proposed by Schwartz (2001)^(2)^, which consists of a personal approach to values, using as a theoretical model the basic human values elucidated by Schwartz himself, and the revised Work-Related Values Scale (EVT-R), which brings the work dimension and has as its theoretical conceptualization the studies of Porto and Pilati (2010)^(3)^.

Using instruments is considered health technology, and the applicability of an instrument can be considered a soft technology^(4)^, as relations of bond production and management are being assessed. Therefore, the application of an instrument was proposed, the Leadership Team Values Assessment (LTVA)^(5)^, which aims to understand individuals’ leadership styles and personal values. LTVA reveals individual leaders’ motivations, understanding which leadership values are important, serving as a guide in decision-making. It is possible, through the results, to understand how leaders currently view the team and where they see opportunities for improvement.

According to Barrett’s model, LTVA is divided into three categories: Foundation; Evolution; and Impact. In addition to categorizing human values into seven levels of consciousness distributed among these categories, it also relates these levels to types of leadership so that each leader tends to focus on a certain area, and their personal values reflect this^(4)^.

Within the Foundation category, there are three types of leadership: crisis management; relationship management; and performance management^(4)^. A crisis management leader knows how to take control when the organization’s viability is under threat, and is committed to the health of the company and employees, the budget, and accounting. A relationship management leader is committed to interpersonal relationships, worrying about maintaining harmonious relationships and good communication with the team. A performance management leader, on the other hand, focuses on performance, the system and processes, and can even become bureaucratic. They care about organization and productivity^(4)^.

In the Impact category, there are also three leadership styles: authentic leadership; mentoring; and visionary leadership. An authentic or inspiring leader is one who is concerned with creating a climate of inspiration/motivation and believes in the values of openness, equality, transparency, trust and commitment. A mentor/partner leader seeks to serve, works alongside their employees, and is empathetic and fair. A visionary leader is one who cares about the world beyond the organization, possessing social responsibility and a sense of sustainability^(4)^.

Another leadership profile is facilitator or innovator, which represents a leader who is always looking for new things, challenges and transformations. This is the type of leadership in the Evolution category^(4)^.

Professor leaders encompass the skills demonstrated by educators who not only teach their students, but also exert influence that extends beyond their own classrooms to other individuals within the educational environment. Within the classroom, professor leaders serve as models of the type of behavior and learning they want to promote in students^(6)^. They are transparent about their own learning process, not hesitating to acknowledge their own mistakes, making their thinking and learning processes visible, allowing students to understand them and follow by example^(7)^.

One can explore the notion that a professor plays the role of knowledge manager, facilitating the authentic encounter between educator and student. The role of an educational leader is extremely important in institutions, as their approach to teaching can directly influence the outcome of the learning process, whether positively or negatively. For this reason, it is essential to reflect on the role of professors, especially in the university context, where their responsibilities are vast and complex. The relationship with students is central, including setting goals, planning actions and designing educational projects. Furthermore, student guidance, especially with regard to motivation and commitment to tasks, is essential. This process aims to create an environment conducive to developing students’ autonomy and confidence, treating them as professionals and encouraging them to explore their maximum potential^(8)^.

It is crucial that professors take aspects related to leadership into consideration as essential elements in their management of the teaching and learning process. In this context, the role of professors as leaders is fundamental. They have the responsibility of encouraging students’ full participation, motivating and inspiring them in their learning process. By doing this, professors not only facilitate student engagement, but also promote an environment of collaboration and mutual growth^(9)^.

The competence of health science professors is comprehensive and multifaceted, due to the complexity of the health area, which requires highly qualified professionals in a context of constant change. There is no universal consensus on the concept of competence, but the main attributes generally include knowledge, skills and attitudes (including values), which interconnect and complement each other in various ways. Health sciences professors are expected not only to possess broad knowledge and pedagogical and research skills, but also competencies in international networking, leadership and management^(10)^.

With the possibility of analyzing individuals’ personal values, the desire to know the values that guide leaders’ behavior arose, since leadership value assessment provides an overview of what moves leaders, how leaders work together and what they want to build or develop for the future. Thus, professors are leaders and have the ability to guide behavior. Therefore, this study had as its guiding question: what are professors’ leadership styles and personal values at a school of nursing?

The justification for the study is to clarify the gap in studies that describe and analyze professors’ motivations and values, especially nursing professors, who are responsible for training future nursing team leaders. Professors nowadays have a complex and broad role. They are no longer transmitters of technical knowledge, but are now considered a guide and influencer of behavior, with a commitment to developing and training professionals in accordance with the professional values that guide the identity of the profession. It is believed that this study will contribute to achieving the Sustainable Development Goals established and proposed by the United Nations (UN) to Brazil^(11)^, in a global action, composed of ambitious and interconnected objectives that address the main development challenges faced by people in Brazil and around the world, such as strengthening education and improving working conditions.

## OBJECTIVES

To identify professors’ leadership styles and personal values at a school of nursing.

## METHODS

### Ethical aspects

The study was approved by the university’s Research Ethics Committee. Professors were invited to participate in the research via email and, if they agreed, they were automatically directed to the Informed Consent Form (ICF), in accordance with Resolution 466/12.

### Study design, period and location

This is a quantitative, descriptive-exploratory study, guided by the STrengthening the Reporting of OBservational studies in Epidemiology (STROBE) tool^(12)^. Data collection took place from June 2021 to August 2021, at a public university in the state of São Paulo, located in the capital.

### Population or sample; inclusion and exclusion criteria

The population consisted of all 13 professors in doctoral 1 category at the school of nursing of a public university. This professional category is the first teaching category in career construction, and the profile is expected to demonstrate engagement in undergraduate teaching activities, converging with the need to develop and improve its way of acting and essence. These professors who had a temporary contract, experts, retirees, professors in doctoral 2 category, associates and full professors, professors who were on vacation or on medical leave or away for another reason during the data collection period were excluded.

### Study protocol

The study population sociodemographic characterization included age, sex and marital status, and professional characterization (time since training, time working as a professor and time held in the position at the current university) was collected via Google Forms^®^. Subsequently, the LTVA^(5)^ instrument was applied through the Barrett Value Center^®^ platform, which made the questionnaire and the results report available in a virtual environment. The LTVA questionnaire consists of three questions, which encompass 37 items related to human values, estimating around 10 to 15 minutes to be answered.

The LTVA identifies seven levels that make up human motivations^(4)^, divided into three categories:

Foundation: The first three levels of consciousness - Viability, Relationships and Performance - focus on foundation, satisfying the need for safety and security, the need for love and belonging, and individuals’ need to feel about themselves through development a sense of pride in being what they are^(4)^.

Evolution: composed of the fourth level of consciousness, Evolution, and focused on Letting Go of Fears. A sense of personal authority and voice is established. Within the Evolution category, one chooses to live in accordance with the values and beliefs that resonate deeply with who we are^(4)^.

Impact: The three higher levels of consciousness - performance, collaboration, and contribution - focus on the need to find meaning and purpose in life. This meaning is expressed by fighting to make the world a better place and living a life of selfless contribution. When these needs are met, they generate deeper levels of motivation and commitment^(4)^.

Chart 1 describes the levels of awareness of leadership styles within the three categories according to Barrett’s model^(5)^.

**Chat 1:** Leadership styles and the seven levels of consciousness, distributed across the three categories, according to the Barrett Model, 2020

Level	Level of consciousness	Types of leadership	Category
7	Contribution	Visionary leader	Impact
6	Collaboration	Mentor/partner leader	
5	Alignment	Authentic leader	
4	Evolution	Facilitator/innovator	Evolution
3	Performance	Performance manager	Foundation
2	Relationship	Relationship manager	
1	Viability	Crisis manager	

*Source: Barrett Value Center®.*

### Analysis of results, and statistics

The LTVA is an ipsative scale, i.e., the scores on a variable depend on the individual level of each person and do not allow interpersonal comparisons to be made, being inappropriate for carrying out statistical analyzes^(13)^. Quantitative data were analyzed using SPSS Statistics 17.0 (2008, SPSS Inc.), using measures of central tendency (mean, standard deviation and variance).

## RESULTS

The population consisted of 13 professors from doctor 1 category at the school of nursing, of which 12 (92.3%) were female, and one (7.69%) was male, as shown in Table 1. It is noteworthy that, of the total number of professors, six (46.15%) have held the position for less than two years, whereas seven professors (53.85%) have held the position of doctor 1 for more than two years and for less than ten years.

**Table 1:** Sociodemographic and occupational data of N=13 professors in doctoral category 1 at the school of nursing of a public university in the state of São Paulo, Brazil, 2021

Characteristics	n	%
Sex		
Female	12	92.30
Male	1	7.69
Marital status		
Married	5	38.46
Divorced	0	0
Single	8	61.54
Stable union	0	0
Occupation time		
Less than six months	0	0
Between six months and one year	1	7.69
Between one and two years	5	38.46
Between two and five years	4	30.77
Between five and ten years	3	23.08
Over ten years	0	0

*Source: survey data.*

The results obtained are compiled in Table 2, according to the LTVA report extracted from the platform.

**Table 2:** Leadership values pointed out by doctor 1 category professors from the school of nursing, considering the level of consciousness, according to the Barrett Model, São Paulo, Brazil, 2021

Leadership values	n^*^	Level
Commitment	7	5
Positive attitude	6	5
Trust	6	5
Accessible	5	2
Inclusion capacity	5	4
Empathy	5	6
Working in collaboration	5	6
Open to new ideas	4	4
Cooperation	4	5
Listening	4	2
Humility	4	7

* *Number of professors.*

*Source: Survey data/Barrett Value Center®.*

It is noteworthy that level 5, which represents authentic leadership, was the most prevalent at 36.4%, with values highlighted in commitment, positive attitude and trust being those highlighted for this level. This level is part of the Impact category. The Impact category also appeared at level 6 (collaboration), which is mentor/partner leader, with 18.2% of personal values found in this leadership style.

Levels 2 and 4, which represent the relationship manager and facilitator/innovator leader, respectively, were also identified in 18.2% of the values each, belonging to the Foundation and Evolution categories.

Level 7, in the Impact category, portrayed by visionary leadership, was the least identified by the population studied at 9% of the total values indicated.

**Figure 1:**
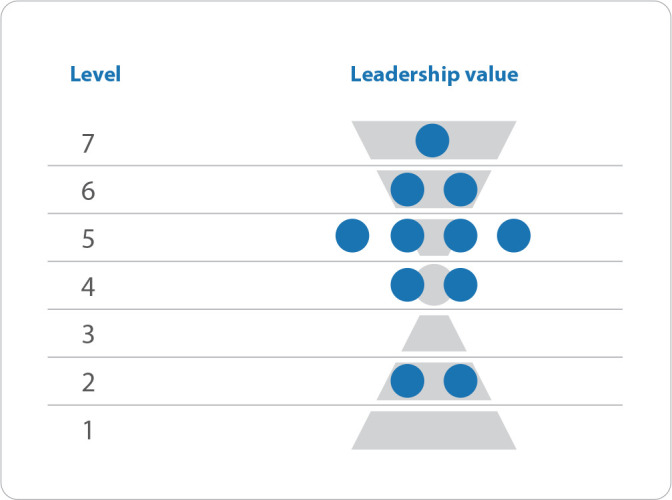
Distribution of leadership values of doctor 1 university professors from the nursing faculty, arranged in the seven levels of leadership consciousness, extracted from the Leadership Team Values Assessment, according to the Barrett Model

*Source: Barrett Value Center^®^.*

Figure 1 demonstrates this distribution of professors’ leadership values between levels of leadership awareness:

## DISCUSSION

Leadership is a complex component and has been increasingly valued in healthcare teaching and recognized as essential for delivering high standards of education, research and clinical practice. Leadership and management skills have been recognized as expectations and requirements in the health education environment in achieving more effective results, since a new type of leader who shapes and performs the balance between autonomy and responsibility emphasizes teamwork and focuses on patient improvement and results, consolidating itself in the healthcare context^(14)^. Mikkonen *et al*. (2018)^(10)^, in their systematic review, highlighted the importance of ethics and leadership as fundamental elements of health professors’ competence, for which knowledge, skills and attitudes related to leadership were considered crucial.

A qualitative study on nursing students’ perception regarding student autonomy promotion was manifested by freedom of action and development of important skills, such as decision-making and leadership, encouraged by the search for a more independent, creative and critical professional profile^(15)^. Still in this study, this teacher is described as having clear aims, purposes, values, and historical and philosophical foundations of professional health education. The idea of solidifying leadership learning through students’ contact with teaching leader models supports the fact that trained and experienced educators are the most suitable individuals for teaching this skill, since the leadership behavior of teaching practice can be reproduced by students^(16)^.

In general, teachers are problem solvers and facilitators, a fundamental role in education. Thus, different leadership styles are employed for classroom management. However, it is extremely important that teachers develop these leadership skills, as this is an essential need to improve the quality of teaching and motivate students to achieve excellent academic results^(17)^.

As observed in the results, the authentic leadership style was the most present, as the personal values presented, such as commitment, positive attitude and trust, were the most prominent. According to the literature, teachers who practice this style present positive results on the part of leaders and followers, as they seek self-development for everyone involved^(18)^. Authentic leaders promote pedagogical practices that activate and offer autonomy to students, enabling them to take a critical look during learning.

Based on the four domains that characterize authentic leader behavior, such as transparency in relationships, moral and ethical perspective, balanced information processing, and self-awareness, authentic leaders design an environment in which followers feel encouraged to express themselves in a genuine and sincere, providing a reduction in negative practices and consolidating group work in the search for the overall effectiveness and success of the organization^(18)^. In this way, authentic leadership can be considered one of the theoretical leadership models most appropriate to the work process developed in the educational system, as it is based on values, character, transparency and ethics^(19)^.

Authentic leadership has been seen as a competent instrument to improve the way students learn in educational institutions, since teachers with this leadership style generate a positive environment, creating a climate of trust, creativity and effectiveness, encouraging the development and good performance of students. Therefore, it can be said that this leadership model is considered the most genuine, positive, transparent and ethical in organizations, including educational institutions^(20)^. Such competence, manifested by a set of skills of teachers who exert influences perceived beyond the classroom, and not only by their institutional position, brings the conception that teachers, as leaders in the teaching context, tend to be the most interfering strong in the learning process^(21)^.

An integrative review pointed out that, in the context of education, authentic leadership is a conceptually new model that has been used in educational management, and has been taught in some undergraduate and graduate courses, generating the need to invest in teaching authentic leadership in this context, since the adoption of this leadership model directly impacts the rates of positive factors related to it, be they trust, involvement, academic optimism, responsibility, creativity, among others^(22)^. This study also revealed that, although this leadership model seeks ethical practices that benefit learning, it is still not a model strongly adopted in education in the area of health sciences, including nursing courses.

The influence of authentic leadership on work in nursing is linked not only to the organization of work, but also to the effectiveness of communication between team members, which requires joint activities of multi-professional cooperation, building relationships of trust and optimism. This influence can improve future nurses’ quality of work and well-being by inspiring a positive, safe, and friendly work environment^(23)^.

Therefore, authentic leadership can positively affect nurses’ attitudes and behaviors, being suitable for promoting expressions of work engagement, organizational citizenship behavior and performance of employees who go above and beyond what is expected in an organization^(24)^.

The least frequently cited leadership style, visionary leadership, is a highly influential leadership style that relies on leaders’ ability to communicate an idealized vision of the future, thereby motivating followers to strive to achieve it^(25)^. Examples of this type of leadership include the ability to clearly establish group direction and regularly communicate how the future should be shaped. The broad positive effects of this approach improve employee organization and commitment as well as job satisfaction, positive emotions, intention to stay and professional performance^(26)^.

Nursing leaders and clinical practice nurses face the challenge of staying ahead of future trends regarding new treatments and need to establish a culture, infrastructure and work environment that encourages innovation, advancement of nursing practice and excellence in person-centered care^(27)^. To achieve these goals, it is essential to incorporate evidence-based practices and use data as fundamental tools. Nurse leaders who support evidence-based practices can promote a culture of inquiry, which lays the foundation for using evidence in leadership and management decision-making. By adopting approaches based on research and proven results, these leaders empower their teams to provide more effective and safe care for patients. By prioritizing the use of evidenceand data-based practices, nursing leaders create an environment conducive to innovation, encouraging the adoption of new approaches and care techniques that can lead to better patient outcomes. Furthermore, this evidence-based approach can strengthen decision-making, making it more informed and informed. In short, the promotion of evidence-based nursing practice by leaders is a fundamental element in driving the quality of care provided and contributing to the continued progress of nursing as a profession. This not only benefits patients, but also strengthens the role of nurses as clinical leaders who constantly strive for excellence in healthcare^(27)^.

The present study also brought relevance to relationship manager and facilitator/innovator, respectively, highlighting the levels of Foundation and Evolution. Considering that evolutional behaviors by teachers deviate from foundation to inspire students and seek their highest levels of functioning, enthusiasm, participation in the classroom, encouraging their critical thinking, a study recently carried out with 3,354 university students in Spain proposed to analyze the influence of teacher leadership on academic resilience and motivation, burnout and academic performance^(28)^. The results observed in this study showed that teacher leadership was positively correlated with academic resilience and motivation. In turn, academic resilience and motivation predicted burnout negatively, whereas academic performance was positively impacted, guiding how academic contexts can influence positive and negative situations in the classroom and how the role of teachers is fundamental to an experience positive feedback from students in this scenario.

Among many responsibilities, nursing professors have a fundamental role in teaching and building leadership skills and constructive conflict management for students. Communication, assertiveness and resilience are essential skills that need to be highlighted in the training of nurses. Clarity and conflict management in the clinical environment can improve nurse-patient relationships, improve nurses’ job satisfaction, and increase the quality of patient care^(29)^. It is known that conflict is inevitable, whether in nursing education or practice. That said, it is important that nursing students are aware of the leadership and conflict management skills necessary to support them in their studies and future careers^(29)^. Leadership and conflict management are linked. In this way, developing appropriate leadership styles and conflict management can positively influence decision-making and therefore potentially improve results in their future activities.

### Study limitations

The limitation of this study is related to the study population consisting only of a hierarchical category of nursing professors within a single public university, not taking into account other categories and other higher education institutions, whether public and/or private. This opens up space for further research, in which it is possible to describe the leadership profile of professors in different scenarios and contexts.

### Contributions to nursing, health, or public policy

The study’s contribution correlates to the search for increasing the quality and efficiency levels of educational institutions through their teaching approaches and practices, since leadership is one of the keys to changing educational systems and organizations. In this scenario, it is suggested that more robust evidence should be developed on professor leadership in the educational system, in order to substantiate the knowledge produced.

## CONCLUSIONS

This study allowed us to verify the importance of reflecting on personal values and how they reflect nursing professors’ leadership styles. This is a period in which student begins to understand what it means to be a nurse and their role in relation to different aspects; therefore, professors can influence and have repercussions on the type of professional they are training. The development of professional values is the responsibility of professors and the university, with the authentic leadership profile being suitable for motivation and to inspire students to believe in and follow the same values, empower them and provide an encouraging and productive learning environment.

It is concluded that professors’ personal values provide a theoretical basis for guiding and analyzing professors’ leadership styles, and the dimensions analyzed are also present in the professor-student relationship. Leadership in the educational context must be widely recognized and studied to promote a more comprehensive and effective approach to developing and improving educational leaders.

The scarcity of scientific studies on the interaction between personal, professional values and leadership is evident. Different leadership styles appear to be influenced by each person’s individual values. However, transforming values is not a trivial task, as it demands a process of deep self-knowledge, continuous personal development and a proactive stance in search of growth. The objective of this process is to become an agent of positive change both in the educational environment and in interpersonal relationships.
