# Supplementary material for: Leadership styles and personal values of professors at a school of nursing
Source: Rev Bras Enferm. 2024 Aug 26;77(5):e20230333. doi: 10.1590/0034-7167-2023-0333 (PMC11346910; doi:10.1590/0034-7167-2023-0333)
Supplement: Translation

# Translation: Estilos de liderança e valores pessoais de docentes universitários de uma faculdade de enfermagem

**DOI:** 10.1590/0034-7167-2023-0333pt

**Authors:** Chennyfer Dobbins Abi Rached, Bianca Batista de Siqueira, Bruna Franco Massa, Caroline da Silva Fonseca Paulo, Sofia de Souza Cruz

**Affiliations:** I Universidade de São Paulo. São Paulo, São Paulo, Brasil

**Keywords:** Liderança, Docentes, Docentes de Enfermagem, Educação, Educação em Enfermagem

## Abstract

**Objetivos:**

identificar os estilos de liderança de docentes universitários e os valores pessoais.

**Métodos:**

estudo quantitativo, descritivo-exploratório. População constituída por docentes universitários da categoria doutor 1 de uma universidade pública do estado de São Paulo. A coleta de dados ocorreu de junho a agosto de 2021. Foi extraída a caracterização sociodemográfica, sendo aplicado o instrumento *Leadership Team Values Assessment*. Os dados foram analisados utilizando medidas de tendência central.

**Resultados:**

população incluiu 13 docentes. O nível que representa a liderança autêntica foi o mais vigente. Os valores comprometimento, atitude positiva e confiança foram destacados. O nível retratado pela liderança visionária foi o menos identificado.

**Conclusões:**

os valores pessoais dos docentes fornecem uma base teórica para pautar e analisar os estilos de liderança de professores. A liderança no contexto educacional deve ser reconhecida e estudada para promover uma abordagem mais abrangente e efetiva no desenvolvimento e aprimoramento de líderes educacionais.

## INTRODUÇÃO

A literatura acadêmica conta com um amplo arcabouço teórico acerca do estudo dos valores humanos. Tal interesse se deve à concepção, reiterada por diversos autores, de que os valores são preditivos do comportamento humano, seja no âmbito pessoal, no coletivo, no familiar, ou no profissional. Portanto, é possível explorar vários aspectos individuais, coletivos e sociais a partir do entendimento dos valores humanos^(1)^.

Os estudos acadêmicos relativos aos valores não se limitaram ao esclarecimento do termo, mas propuseram formas de avaliação de valores, visto que a análise dos valores pessoais de um líder diz muito sobre suas motivações e seu estilo de liderança. Destacam-se algumas escalas validadas para avaliação de valores, como a *Portrait Questionnaire Value* (PQV), proposta por Schwartz (2001)^(2)^, que consiste em uma abordagem pessoal dos valores, tendo como modelo teórico os valores humanos básicos elucidados pelo próprio Schwartz, e a Escala revisada de Valores relativos ao Trabalho (EVT-R), a qual traz a dimensão do trabalho e tem como conceituação teórica os estudos de Porto e Pilati (2010)^(3)^.

A utilização de instrumentos é considerada tecnologia em saúde, e a aplicabilidade de um instrumento pode ser considerada uma tecnologia leve^(4)^, já que estão sendo avaliadas relações de produção de vínculo e gestão. Sendo assim, foi proposta a aplicação de um instrumento, o *Leadership Team Values Assessment* (LTVA)^(5)^, que se dispõe a compreender os estilos de liderança e valores pessoais dos indivíduos. O LTVA revela as motivações de líderes individuais, entendendo quais valores de liderança são importantes, servindo como guia na tomada de decisão. É possível, por meio dos resultados, entender como os líderes atualmente visualizam a equipe e onde eles veem oportunidades de melhoria.

Segundo o modelo de Barrett, o LTVA é dividido em três categorias: Fundação; Evolução; e Impacto. Além de categorizar os valores humanos em sete níveis de consciência distribuídos entre essas categorias, também relaciona esses níveis com tipos de liderança, de modo que cada líder costuma focar-se em uma determinada área, e seus valores pessoais refletem isso^(4)^.

Dentro da categoria Fundação, há três tipos de liderança: gerenciamento de crises; gerenciamento de relacionamentos; e gerenciamento de *performance* ^(4)^. Um líder gerenciador de crises sabe assumir o controle quando a viabilidade da organização fica sob ameaça, e se empenha na saúde da empresa e dos funcionários, no orçamento, na contabilidade. Um líder gerenciador de relacionamentos empenha-se nas relações interpessoais, preocupando-se em manter as relações harmoniosas e uma boa comunicação com a equipe. Já o líder gerente de *performance* concentra-se na *performance*, no sistema e nos processos, podendo tornar-se inclusive burocrático. Eles se preocupam com a organização e produtividade^(4)^.

Na categoria Impacto, também há três estilos de liderança: liderança autêntica; mentoria; e liderança visionária. O líder autêntico ou inspirador é aquele que se preocupa em criar um clima de inspiração/motivação e acredita nos valores de abertura, igualdade, transparência, confiança e comprometimento. O líder mentor/parceiro busca servir, se coloca junto com seus colaboradores, é empático e justo. O líder visionário é aquele que se preocupa com o mundo para além da organização, possuindo reponsabilidade social e senso de sustentabilidade^(4)^.

Outro perfil de liderança é a facilitadora ou inovadora, que representa o líder que está sempre em busca do novo, de desafios e de transformações. Esse é o tipo de liderança da categoria Evolução^(4)^.

Líderes docentes englobam as habilidades demonstradas por educadores que não só ensinam seus alunos, mas também exercem influência que se estende para além de suas próprias salas de aula, alcançando outros indivíduos dentro do ambiente educacional. Dentro da sala de aula, os professores líderes servem como modelos do tipo de comportamento e aprendizado que desejam promover nos alunos^(6)^. Eles são transparentes em relação ao seu próprio processo de aprendizagem, não hesitando em reconhecer seus próprios erros, tornando seus processos de pensamento e aprendizado visíveis, permitindo que os alunos os compreendam e sigam como exemplo^(7)^.

Pode-se explorar a noção de que o professor desempenha o papel de gestor do conhecimento, facilitando o encontro autêntico entre educador e aluno. O papel do líder educacional é de extrema importância nas instituições, pois sua abordagem na condução da docência pode influenciar diretamente o resultado do processo de aprendizagem, seja de forma positiva ou negativa. Por esse motivo, é essencial refletir sobre a figura do professor, especialmente no contexto universitário, onde suas responsabilidades são vastas e complexas. O relacionamento com os alunos é central, incluindo o estabelecimento de metas, o planejamento de ações e a concepção de projetos educacionais. Além disso, a orientação dos alunos, especialmente no que diz respeito à motivação e ao comprometimento com as tarefas, é fundamental. Esse processo visa criar um ambiente propício ao desenvolvimento da autonomia e da confiança dos alunos, tratando-os como profissionais e incentivando-os a explorar seu potencial máximo^(8)^.

É crucial que o professor leve em consideração aspectos relacionados à liderança como elementos essenciais em seu manejo do processo de ensino e aprendizagem. Nesse contexto, o papel do professor como líder é fundamental. Ele tem a responsabilidade de incentivar a participação plena dos alunos, motivando-os e inspirando-os em seu processo de aprendizagem. Ao fazer isso, o professor não apenas facilita o engajamento dos estudantes, mas também promove um ambiente de colaboração e crescimento mútuo^(9)^.

A competência dos professores de ciências da saúde é abrangente e multifacetada, devido à complexidade da área da saúde, que requer profissionais altamente qualificados em um contexto de constante mudança. Não há um consenso universal sobre o conceito de competência, mas os principais atributos geralmente incluem conhecimento, habilidades e atitudes (incluindo valores), os quais se interconectam e se complementam de várias maneiras. Professores de ciências da saúde são esperados não apenas a possuir amplo conhecimento e habilidades pedagógicas e de pesquisa, mas também competências em *networking* internacional, liderança e gestão^(10)^.

Com a possibilidade de analisar valores pessoais de indivíduos, surgiu o anseio de conhecer os valores que norteiam o comportamento de líderes, posto que a avaliação de valores de liderança fornece uma visão geral do que move o líder, como os líderes trabalham juntos e o que desejam construir ou desenvolver para o futuro. Nesse sentido, docentes são líderes e possuem a capacidade de orientar comportamentos. Logo, este estudo teve como pergunta norteadora: quais os estilos de liderança e valores pessoais de docentes universitários de uma faculdade de enfermagem?

A justificativa do estudo consiste em esclarecer a lacuna existente acerca de estudos que descrevam e analisem as motivações e valores de professores universitários, sobretudo os de enfermagem, que possuem a responsabilidade de formação de futuros líderes da equipe de enfermagem. O docente nos dias de hoje tem um papel complexo e amplo. Ele deixou de ser o transmissor de conhecimento técnico, passando a ser considerado orientador e influenciador de comportamento, tendo o compromisso de desenvolver e formar profissionais de acordo com os valores profissionais que norteiam a identidade da profissão. Acredita-se que este estudo irá contribuir com o alcance das metas dos Objetivos de Desenvolvimento Sustentável estabelecidos e propostos pela Organização das Nações Unidas (ONU) ao Brasil^(11)^, em uma ação global, compostos de objetivos ambiciosos e interconectados que abordam os principais desafios de desenvolvimento enfrentados por pessoas no Brasil e no mundo, como o fortalecimento da educação e a melhoria das condições de trabalho.

## OBJETIVOS

Identificar os estilos de liderança de docentes universitários de uma faculdade de enfermagem e os valores pessoais.

## MÉTODOS

### Aspectos éticos

O estudo passou pela aprovação do Comitê de Ética em Pesquisa da universidade. Os docentes foram convidados a participar da pesquisa via e-mail e, estando de acordo, foram direcionados automaticamente para o Termo de Consentimento Livre e Esclarecido (TCLE), de acordo com a Resolução n^o^. 466/12.

### Desenho, período e local do estudo

Trata-se de estudo quantitativo, descritivo-exploratório, norteado pela ferramenta *STrengthening the Reporting of OBservational studies in Epidemiology* (STROBE)^(12)^. A coleta de dados ocorreu de junho de 2021 a agosto de 2021, em uma universidade pública do estado de São Paulo, localizada na capital de mesmo nome.

### População ou amostra; critérios de inclusão e exclusão

A população foi constituída por todos os 13 docentes universitários da categoria doutor 1 da faculdade de enfermagem de uma universidade pública. Essa categoria profissional é a primeira categoria docente na construção da carreira, sendo o perfil esperado demonstrar o engajamento nas atividades de ensino da graduação, convergindo com a necessidade de desenvolvimento e aprimoramento da sua forma de atuação e essência. Esses docentes que possuíam regime de contratação temporário, especialistas, aposentados, professores das categorias doutor 2, associados e titulares, professores que estavam de férias ou em licença médica ou afastados por outro motivo durante o período de coleta dos dados foram excluídos.

### Protocolo do estudo

A caracterização sociodemográfica da população do estudo incluiu idade, sexo e estado civil, e a caracterização profissional (tempo de formação, tempo de trabalho como docente e tempo de ocupação no cargo na universidade atual) foi coletada via *Google Forms* ^®^. Em seguida, foi aplicado o instrumento LTVA^(5)^, por meio da plataforma Barrett Value Center^®^, que disponibilizou em ambiente virtual o questionário e também o relatório de resultados. O questionário do LTVA é composto de três perguntas, as quais englobam 37 itens relacionados a valores humanos, estimando-se em torno de 10 a 15 minutos para ser respondido.

O LTVA identifica sete níveis que compõem as motivações humanas^(4)^, divididas em três categorias:

Fundação: os primeiros três níveis de consciência - viabilidade, relacionamentos e desempenho - se concentram em fundação, satisfazer a necessidade de segurança e proteção, necessidade de amor e de pertencer, e necessidade do indivíduo sentir-se consigo mesmo através do desenvolvimento de uma sensação de orgulho em ser o que é^(4)^.

Evolução: composta pelo quarto nível de consciência, evolução, e focada em abrir mão dos medos. Estabelece-se uma noção da própria autoridade pessoal e da própria voz. Dentro da categoria Evolução, escolhe-se viver de acordo com os valores e crenças que ressoam profundamente com quem somos^(4)^.

Impacto: os três níveis superiores de consciência - alinhamento, colaboração e contribuição - se concentram na necessidade de encontrar significado e propósito na vida. Expressa-se esse significado lutando para tornar o mundo um lugar melhor e levando uma vida de serviço abnegado. Quando essas necessidades são atendidas, geram níveis mais profundos de motivação e compromisso^(4)^.

O Quadro1 descreve os níveis de consciência aos estilos de liderança dentro das três categorias, de acordo com o modelo de Barrett^(5)^.

**Quadro 1:** Estilos de liderança e os sete níveis de consciência, distribuídos nas três categorias, de acordo com o Modelo de Barrett, 2020

Nível	Nível de consciência	Tipos de liderança	Categoria
7	Contribuição	Líder visionário	Impacto
6	Colaboração	Líder mentor/parceiro	
5	Alinhamento	Líder autêntico	
4	Evolução	Facilitador/inovador	Evolução
3	Desempenho	Gestor de desempenho	Fundação
2	Relacionamento	Gerente de relacionamento	
1	Viabilidade	Gestor de crises	

*Fonte: Barrett Value Center®.*

### Análise dos resultados e estatística

O LTVA é uma escala ipsativa, ou seja, as pontuações em uma variável dependem do nível individual de cada um e não permitem que sejam realizadas comparações interpessoais, sendo inapropriadas para realização de análises estatísticas^(13)^. Os dados quantitativos foram analisados através do SPSS *Statistics* 17.0 (2008, SPSS Inc.), utilizando-se medidas de tendência central (média, desvio padrão e variância).

## RESULTADOS

A população foi constituída pelos 13 professores universitários da categoria doutor 1 da faculdade de enfermagem, dos quais 12 (92,3%) eram do sexo feminino, e um (7,69%), do sexo masculino, conforme demonstrado na Tabela 1. Destaca-se que, do total de professores, seis (46,15%) ocupam o cargo há menos de dois anos, enquanto que sete professores (53,85%) ocupam o cargo de doutor 1 há mais de dois e há menos de dez anos.

**Tabela 1:** Dados sociodemográficos e ocupacionais dos N=13 professores universitários da categoria doutor 1 da faculdade de enfermagem de uma universidade pública do estado de São Paulo, Brasil, 2021

Características	n	%
Sexo		
Feminino	12	92,30
Masculino	1	7,69
Estado civil		
Casado	5	38,46
Divorciado	0	0
Solteiro	8	61,54
União estável	0	0
Tempo de ocupação		
Menos que seis meses	0	0
Entre seis meses e um ano	1	7,69
Entre um e dois anos	5	38,46
Entre dois e cinco anos	4	30,77
Entre cinco e dez anos	3	23,08
Acima de dez anos	0	0

Os resultados obtidos estão compilados na Tabela 2, conforme o relatório do LTVA extraído da plataforma:

**Tabela 2:** Valores de liderança apontados pelos docentes universitários categoria doutor 1 da faculdade de enfermagem, considerando o nível de consciência, de acordo com o Modelo de Barrett, São Paulo, Brasil, 2021

Valores de liderança	n^*^	Nível
Comprometimento	7	5
Atitude positiva	6	5
Confiança	6	5
Acessível	5	2
Capacidade de inclusão	5	4
Empatia	5	6
Trabalhar em colaboração	5	6
Aberto a novas ideias	4	4
Cooperação	4	5
Escutar	4	2
Humildade	4	7

* *Número de docentes.*

*Fonte: Barrett Value Center^®^.*

Destaca-se que o nível 5, que representa a liderança autêntica, foi o mais vigente em 36,4%, sendo que os valores apontados em comprometimento, atitude positiva e confiança foram os destacados para esse nível. Esse nível faz parte da categoria Impacto. A categoria Impacto também apareceu no nível 6 (colaboração), que se trata do líder mentor/parceiro, sendo que 18,2% dos valores pessoais encontram-se nesse estilo de liderança.

Os níveis 2 e 4, que representam o gestor de relacionamento e o facilitador/inovador, respectivamente, também foram apontados em 18,2% dos valores cada um, pertencentes às respectivas categorias Fundação e Evolução.

O nível 7, da categoria Impacto, retratado pela liderança visionária, foi o menos identificado pela população estudada em 9% do total dos valores apontados.

A Figura 1 demonstra essa distribuição dos valores de liderança dos docentes entre os níveis de consciência da liderança:

**Figura 1:** Distribuição dos valores de liderança dos docentes universitários doutor 1 da faculdade de enfermagem, dispostos nos sete níveis de consciência da liderança, extraídos do *Leadership Team Values Assessment*, de acordo com o Modelo de Barrett

*Fonte: Barrett Value Center^®^.*

## DISCUSSÃO

A liderança é um componente complexo e tem sido cada vez mais valorizado na docência em saúde e reconhecido como essencial para a entrega de altos padrões de educação, pesquisa e prática clínica. Habilidades em liderança e gerenciamento têm sido reconhecidas como expectativas e exigências no ambiente de educação em saúde no alcance de resultados mais eficazes, visto que um novo tipo de líder que molda e desempenha o equilíbrio entre autonomia e responsabilidade enfatiza o trabalho em equipe e se concentra na melhoria e resultados do paciente, consolidando-se no contexto em saúde^(14)^. Mikkonen *et al*. (2018)^(10)^, em sua revisão sistemática, destacaram a importância da ética e da liderança como elementos fundamentais da competência de professores da área da saúde, para a qual conhecimentos, habilidades e atitudes relacionadas à liderança foram considerados cruciais.

Estudo qualitativo sobre a percepção de estudantes de enfermagem acerca da promoção da autonomia do discente se manifestou pela liberdade de ação e desenvolvimento de importantes habilidades, como tomada de decisão e liderança, incentivados pela busca de um perfil profissional mais independente, criativo e crítico^(15)^. Ainda neste estudo, esse docente é descrito por ter fins claros, propósitos, valores, e fundamentos históricos e filosóficos da educação profissional em saúde. A ideia da solidificação do aprendizado da liderança por meio do contato do discente com modelos de líderes docentes corrobora o fato de educadores, capacitados e experientes, serem os indivíduos mais adequados para o ensino dessa competência, uma vez que o comportamento de liderança da prática docente pode ser reproduzido pelo estudante^(16)^.

Em geral, docentes são solucionadores de problemas e facilitadores, papel fundamental na educação. Assim, diversos estilos de liderança são empregados para a gestão de sala de aula. No entanto, é de extrema importância que os professores desenvolvam essas habilidades de liderança, uma vez que essa é uma necessidade essencial para melhorar a qualidade da docência e motivar os estudantes a alcançar resultados acadêmicos de excelência^(17)^.

Conforme observado nos resultados, o estilo de liderança autêntica foi o mais presente, visto que os valores pessoais apresentados, como comprometimento, atitude positiva e confiança, foram os mais destacados. Segundo a literatura, docentes que exercem esse estilo apresentam resultados positivos por parte dos líderes e liderados, pois buscam o autodesenvolvimento de todos os envolvidos^(18)^. Líderes autênticos promovem práticas pedagógicas que ativam e ofertam autonomia ao discente, fazendo com que sejam capazes de ter um olhar crítico durante o aprendizado.

Baseado nos quatro domínios que caracterizam o comportamento do líder autêntico, como transparência nos relacionamentos, perspectiva moral e ética, processamento equilibrado de informações, e autoconsciência, o líder autêntico projeta um ambiente no qual os liderados sentem-se estimulados a se autoexpressar de maneira genuína e sincera, proporcionando redução em práticas negativas e consolidando o trabalho em grupo na busca pela eficácia geral e sucesso da organização^(18)^. Dessa forma, a liderança autêntica pode ser considerada um dos modelos teóricos de liderança mais adequados ao processo de trabalho desenvolvido no sistema educacional, visto que se baseia nos valores, no caráter, na transparência e na ética^(19)^.

A liderança autêntica tem sido encarada como um instrumento competente para melhorar a forma de aprendizagem entre os estudantes em instituições educacionais, uma vez que docentes com esse estilo de liderança geram um ambiente positivo, criam um clima de confiança, criatividade e eficácia, incentivando o desenvolvimento e bom desempenho dos discentes. Sendo assim, pode-se dizer que esse modelo de liderança é considerado o mais genuíno, positivo, transparente e ético nas organizações, incluindo as instituições de ensino^(20)^. Tal competência, manifestada por um conjunto de habilidades de professores que exercem influências percebidas para além da sala de aula, e não somente por sua posição institucional, traz a concepção de que o docente, enquanto líder no contexto do ensino, tende a ser a interferência mais forte no processo de aprendizagem^(21)^.

Revisão integrativa apontou que, no âmbito da educação, a liderança autentica é um modelo conceitualmente novo que tem sido utilizado na gestão educacional, e tem sido ensinado em alguns cursos de graduação e pós-graduação, gerando a necessidade em se investir no ensino da liderança autentica nesse contexto, uma vez que a adoção desse modelo de liderança impacta diretamente os índices de fatores positivos a ela relacionadas, sejam eles confiança, envolvimento, otimismo acadêmico, responsabilidade, criatividades, entre outros^(22)^. Este estudo também revelou que, apesar desse modelo de liderança buscar práticas éticas que beneficiam o aprendizado, ele ainda não constitui um modelo fortemente adotado na educação na área das ciências da saúde, incluindo os cursos de enfermagem.

A influência da liderança autêntica sobre o trabalho no âmbito da enfermagem está ligada não só à organização do trabalho, mas também à efetividade da comunicação entre os membros da equipe, a qual exige atividades em conjunto de cooperação multiprofissional, construindo relacionamentos de confiança e otimismo. Essa influência pode melhorar a qualidade do trabalho e o bem-estar dos futuros enfermeiros, ao inspirar um ambiente de trabalho positivo, seguro e amigável^(23)^.

Portanto, a liderança autêntica pode atingir positivamente as atitudes e os comportamentos dos enfermeiros e enfermeiras, sendo adequada para promover expressões de engajamento no trabalho, comportamento de cidadania organizacional e *performance* de funcionários que fazem além do esperado em uma organização^(24)^.

O estilo de liderança menos apontado, a liderança visionária, é um estilo de liderança altamente influente que se baseia na capacidade do líder de comunicar uma visão idealizada do futuro, motivando, assim, seus seguidores a se empenhar para alcançá-la^(25)^. Exemplos desse tipo de liderança incluem a habilidade de estabelecer claramente a direção do grupo e comunicar regularmente como o futuro deve ser moldado. Os amplos efeitos positivos dessa abordagem aprimoram a organização e o comprometimento dos colaboradores, bem como sua satisfação no trabalho, emoções positivas, intenção de permanência e desempenho profissional^(26)^.

Os líderes de enfermagem e enfermeiros da prática clínica enfrentam o desafio de estar à frente das tendências futuras em relação aos novos tratamentos e precisam estabelecer uma cultura, infraestrutura e ambiente de trabalho que incentivem a inovação, o avanço da prática de enfermagem e a excelência no cuidado centrado na pessoa^(27)^. Para atingir esses objetivos, é essencial incorporar práticas baseadas em evidências e utilizar dados como ferramentas fundamentais. Os líderes de enfermagem que apoiam práticas baseadas em evidências podem promover uma cultura de investigação, o que estabelece a base para o uso das evidências na tomada de decisões de liderança e gestão. Ao adotar abordagens embasadas em pesquisas e resultados comprovados, esses líderes capacitam suas equipes a proporcionar um cuidado mais eficaz e seguro aos pacientes. Ao priorizar a utilização de práticas baseadas em evidências e dados, os líderes de enfermagem criam um ambiente propício à inovação, incentivando a adoção de novas abordagens e técnicas de cuidado que possam levar a melhores resultados para os pacientes. Além disso, essa abordagem baseada em evidências pode fortalecer a tomada de decisões, tornando-a mais informada e fundamentada. Em suma, a promoção da prática de enfermagem baseada em evidências por parte dos líderes é um elemento fundamental para impulsionar a qualidade do cuidado oferecido e contribuir para o progresso contínuo da enfermagem como profissão. Isso não apenas beneficia os pacientes, mas também fortalece o papel dos enfermeiros como líderes clínicos que buscam constantemente a excelência no cuidado de saúde^(27)^.

O presente estudo também trouxe relevância ao gestor de relacionamento e ao facilitador/inovador, respectivamente, evidenciando os níveis de Fundação e Evolução. Considerando que os comportamentos transformacionais exibidos pelos docentes se desviam do fundação para inspirar os estudantes e buscar seus níveis mais elevados de funcionamento, entusiasmo, participação na sala de aula, encorajando seu pensamento crítico, estudo realizado recentemente com 3.354 estudantes universitários na Espanha propôs analisar a influência da liderança do docente sobre resiliência acadêmica e motivação, *burnout* e desempenho acadêmico^(28)^. Os resultados observados neste estudo mostraram que a liderança do docente se correlacionou positivamente com resiliência acadêmica e motivação. Por sua vez, a resiliência acadêmica e a motivação previram o *burnout* negativamente, enquanto que o desempenho acadêmico foi impactado positivamente, norteando como os contextos acadêmicos podem influenciar situações positivas e negativas em sala de aula e como o papel do professor é fundamental para uma experiência positiva dos discentes nesse cenário.

Entre muitas atribuições, os docentes de enfermagem têm um papel fundamental no ensino e na construção de habilidades de liderança e gestão construtiva de conflitos aos discentes. Comunicação, assertividade e resiliência são habilidades essenciais que precisam ser ressaltadas na formação do enfermeiro. A lucidez e o gerenciamento de conflitos no ambiente clínico podem melhorar as relações enfermeiro-paciente, melhorar satisfação dos enfermeiros no trabalho e aumentar a qualidade do atendimento ao paciente^(29)^. Sabidamente, o conflito é inevitável, seja na educação ou prática de enfermagem. Posto isso, é importante que os estudantes de enfermagem tenham ciência sobre as habilidades em liderança e gestão de conflitos necessária para apoiá-los em seus estudos e futuras carreiras^(29)^. Liderança e gestão de conflitos estão ligadas. Dessa forma, o desenvolvimento de estilos apropriados de liderança e a gestão de conflitos podem influenciar positivamente a tomada de decisão e, portanto, potencialmente melhorar os resultados em suas atividades futuras.

### Limitações do estudo

A limitação deste estudo se relaciona à população do estudo constituída apenas de uma categoria hierárquica de docentes universitários de enfermagem dentro de uma única universidade pública, não levando em consideração outras categorias e outras instituições de ensino superior, sejam elas públicas e/ou privadas. Dessa forma, abre-se um espaço para mais pesquisas, nas quais seja possível descrever o perfil de liderança de docentes em diferentes cenários e contextos.

### Contribuições para a área da enfermagem, saúde, ou políticas públicas

A contribuição do estudo correlaciona-se à busca pelo aumento dos níveis de qualidade e eficiência das instituições de ensino por meio de suas abordagens e práticas docentes, uma vez que a liderança é uma das chaves para a mudança dos sistemas educativos e das organizações. Neste cenário, sugere-se que evidências mais robustas devem ser desenvolvidas na temática da liderança dos docentes no sistema educacional, no intuito de substanciar o conhecimento produzido.

## CONCLUSÕES

Este estudo permitiu verificar a importância de refletirmos sobre os valores pessoais e como eles refletem os estilos de liderança do docente do curso de graduação em enfermagem. Trata-se de um período em que o discente começa a compreender o que é ser enfermeiro e o seu papel em relação a diversos aspectos; portanto, o docente pode influenciar e repercutir sobre o tipo de profissional que ele está formando. O desenvolvimento dos valores profissionais é responsabilidade do professor e da universidade, sendo o perfil de liderança autêntica adequado para motivação e para inspirar os estudantes a acreditar e seguir os mesmos valores, capacitá-los e proporcionar um ambiente de aprendizado estimulante e produtivo.

Conclui-se que os valores pessoais dos docentes fornecem uma base teórica para pautar e analisar os estilos de liderança de professores, sendo que as dimensões analisadas também estão presentes na relação professor-estudante. A liderança no contexto educacional deve ser amplamente reconhecida e estudada para promover uma abordagem mais abrangente e efetiva no desenvolvimento e aprimoramento de líderes educacionais.

A escassez de estudos científicos sobre a interação entre valores pessoais, profissionais e liderança é evidente. Os diferentes estilos de liderança parecem ser influenciados pelos valores individuais de cada pessoa. No entanto, a transformação dos valores não é uma tarefa trivial, pois ela demanda um processo de autoconhecimento profundo, desenvolvimento pessoal contínuo e uma postura proativa em busca do crescimento. O objetivo desse processo é se tornar um agente de mudança positiva tanto no ambiente educacional quanto nas relações interpessoais.
